# Determinants of clinical outcome after transarterial embolization for nonvariceal gastrointestinal bleeding: a retrospective cohort study

**DOI:** 10.1186/s12893-026-03509-8

**Published:** 2026-01-19

**Authors:** Dávid Ádám Korda, Nguyen Tin Dat, András Bibok, Dénes Balázs Horváthy, Ádám Zoltán Farkas, Szabolcs Takács, István Hritz, Attila Szijártó, Bánk Gábor Fenyves, Pál Ákos Deák

**Affiliations:** 1 Department of Interventional Radiology, Semmelweis University, Határőr út 18, Budapest, H-1122 Hungary; 2 Károli Gáspár University of the Reformed Church in Hungary, Kálvin Square 9, Budapest, H-1091 Hungary; 3 Department of Surgery, Transplantation and Gastroenterology, Semmelweis University, Üllői út 78, Budapest, H-1082 Hungary; 4 Department of Emergency Medicine, Semmelweis University, Üllői út 26, Budapest, H-1085 Hungary

**Keywords:** Transarterial embolization, Gastrointestinal bleeding, Clinical success, 30-day mortality

## Abstract

**Background:**

Transarterial embolization (TAE) is an established therapeutic option for non-variceal gastrointestinal bleeding (GIB), but standardized criteria for patient selection and predictors of treatment outcomes remain limited. This study aimed to evaluate clinical outcomes after TAE and identify factors associated with treatment failure and 30-day mortality.

**Methods:**

We retrospectively reviewed all TAE procedures performed for nonvariceal GIB at Semmelweis University between May 2022 and July 2025. Clinical, laboratory, and procedural parameters were collected, including bleeding location, comorbidity burden, antithrombotic therapy, transfusion requirements, vasopressor or inotropic support, and embolization technique. Predictors of clinical failure (rebleeding within 30 days) and 30-day mortality were assessed using multivariable logistic regression.

**Results:**

A total of 111 embolizations were performed in 100 patients. Technical success was 100%, and clinical success was achieved in 82% of patients. The 30-day all-cause mortality rate was 26%, with disease-specific mortality at 15%. Complications occurred in 4% of cases. Vasopressor or inotropic therapy and antithrombotic use were independent predictors of clinical failure. Vasopressor requirement and higher Charlson Comorbidity Index (CCI) scores were significantly associated with 30-day mortality. Empiric embolization was associated with a lower likelihood of rebleeding but did not influence mortality.

**Conclusions:**

TAE provided high technical success and favorable overall outcomes with a low complication rate in the management of nonvariceal GIB. Vasopressor requirement was the strongest predictor of both rebleeding and mortality, likely reflecting the underlying severity of shock physiology rather than the direct effects of vasopressor therapy. These results highlight the need for further prospective studies to guide management strategies in hemodynamically unstable patients requiring vasopressor support.

## Background

Gastrointestinal bleeding (GIB) is a complex clinical condition that requires a multidisciplinary approach. In recent decades, transarterial embolization (TAE) has become the third cornerstone of GIB management, complementing endoscopy and surgery.

For upper gastrointestinal bleeding (UGIB), current guidelines recommend endoscopy as the primary treatment modality [1, 2]. Despite advances in endoscopic therapy, rebleeding occurs in 13–17% of patients and is associated with a substantial increase in mortality risk [3]. Patients with recurrent bleeding after initial endoscopic therapy should undergo repeat endoscopy. If bleeding persists or recurs despite a second attempt, TAE or surgical intervention is indicated. The American College of Gastroenterology (ACG) favors TAE over surgery due to its lower complication rates and shorter hospital stays [1, 4, 5].

The management of lower gastrointestinal bleeding (LGIB) is primarily guided by hemodynamic status. In unstable patients with ongoing hematochezia, computed tomography angiography (CTA) is recommended as the first-line diagnostic tool. A positive CTA should prompt angiography for potential embolization. Colonoscopy may be considered optionally depending on patient stability and institutional expertise, while surgery is reserved for cases refractory to both endoscopic and interventional management [6, 7].

Although TAE is widely used in the management of nonvariceal GIB, evidence regarding predictors of clinical success remains limited, and reported outcomes vary considerably across studies. The relative contributions of patient characteristics, bleeding etiology, and procedural factors to treatment failure and short-term mortality are not well defined. These gaps hinder consistent patient selection, risk stratification, and the development of standardized management pathways.

This study aims to evaluate the outcomes of TAE for nonvariceal UGIB and LGIB and to identify clinical and procedural factors associated with treatment failure and 30-day mortality.

## Methods

### Patient selection

Between May 2022 and July 2025, all patients who underwent TAE for nonvariceal GIB at the Department of Interventional Radiology, Semmelweis University, Budapest, Hungary, were retrospectively identified.

The decision to proceed with TAE was made by a multidisciplinary team, including the on-call interventional radiologist, gastroenterologist, and attending surgeon, in accordance with routine clinical practice. TAE was indicated in the following situations:

1. *UGIB*: after failed endoscopic therapy, in cases of recurrent bleeding following a second endoscopic attempt, or when endoscopic treatment was not feasible.
2. *LGIB*: patients with hemodynamically significant hematochezia underwent CTA; those with contrast extravasation or indirect signs of bleeding were referred for TAE.

Coagulation status was assessed and optimized prior to all procedures. An international normalized ratio (INR) < 2.0 and platelet count > 50,000/µL were considered acceptable thresholds, consistent with contemporary clinical practice recommendations [8, 9]. When values were outside these ranges, correction was performed when clinically appropriate using platelet transfusions, fresh frozen plasma, or factor concentrates (e.g., prothrombin complex concentrate) before embolization. In cases of severe, uncorrectable coagulopathy or persistent hemodynamic instability despite adequate resuscitation, the risks and benefits of proceeding with TAE were carefully evaluated. In our institutional practice, such scenarios were considered relative contraindications, and TAE was deferred when the anticipated procedural risk outweighed the potential therapeutic benefit.

During the study period, a total of 111 embolization procedures were performed in 100 patients, including 11 repeat embolizations in 10 patients. Patients evaluated for angiography but not treated with TAE were not included in the cohort.

### Procedural details

 Informed consent was obtained from all patients prior to the procedure. Electrocardiography, blood pressure, and oxygen saturation were continuously monitored during the procedures. Prior to each intervention, endoscopic reports and contrast-enhanced computed tomography (CTA) images were carefully reviewed.

The right common femoral artery (CFA) was used for vascular access in most cases. A 4- or 5-F vascular sheath was inserted using the Seldinger technique. The visceral arteries were selectively cannulated using appropriate catheters, including Cobra C2, Uni Select 2, and Simmons 1 from the femoral access; and Headhunter 1 and Judkins Right 4 from the brachial approach (Cordis, Santa Clara, CA, USA). The appropriate visceral artery was selected based on pretreatment CTA, endoscopic reports, and, when available, endoscopic clips.

**Fig. 1:**
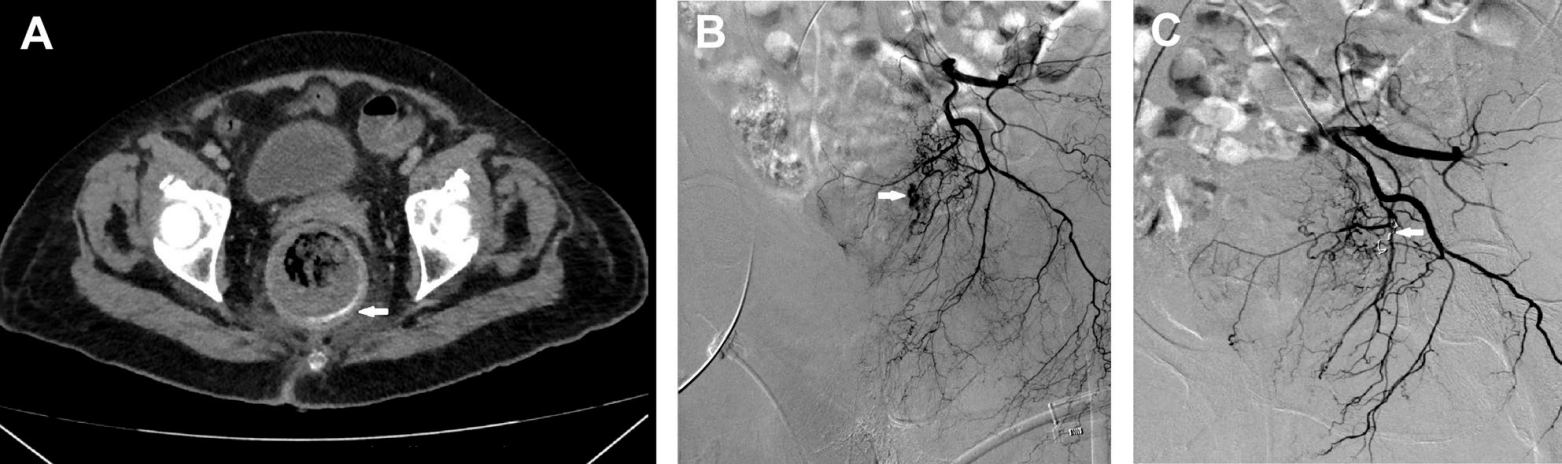
Superselective coil embolization of a middle rectal artery branch in a patient with LGIB. **A** Late-phase CTA image showing contrast pooling (white arrow) in the rectal lumen. **B** Left internal iliac artery angiogram demonstrating contrast extravasation (white arrow) from a middle rectal artery branch. **C** Control angiogram after superselective catheterization and coil embolization (white arrow) of the affected branch, showing no signs of bleeding

**Fig. 2:**
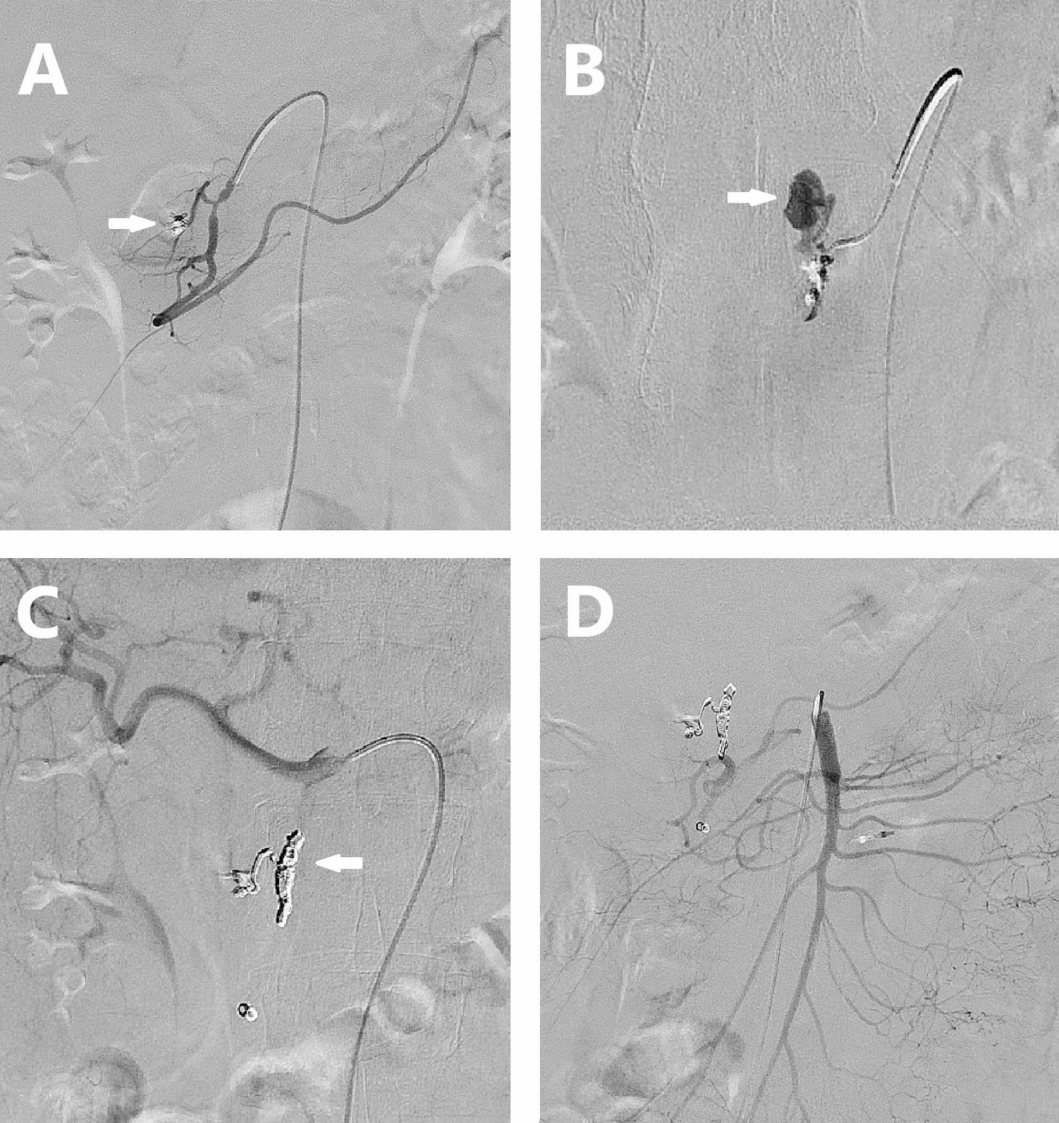
Frontdoor-backdoor coil embolization of the GDA in a patient with persistent UGIB after endoscopic therapy. **A** GDA angiogram showing no apparent bleeding, with endoscopic clips (white arrow) visible in the region of a pancreaticoduodenal branch. **B** Superselective angiogram of the branch leading to the endoscopic clips revealing brisk contrast extravasation (white arrow). **C** Control angiogram of the common hepatic artery after coil embolization (white arrow) showing no signs of bleeding. **D** SMA control angiogram confirming the absence of bleeding

Selective visceral angiography was performed using a power injector. A 1.7–2.7-F microcatheter (Terumo Progreat, Terumo Corporation, Tokyo, Japan; Merit Maestro, Merit Medical, South Jordan, Utah, USA.) was then advanced as close to the bleeding site as technically feasible (Fig. 1). In the gastroduodenal artery (GDA) territory, where collaterals are usually present, frontdoor-backdoor embolization was performed whenever possible (Fig. 2). When no extravasation or indirect signs of bleeding were evident on selective or superselective angiography, the operator assessed the risks and benefits of proceeding with empiric embolization.

The choice of embolic material was determined by operator preference, taking into account bleeding location and underlying etiology. As a general rule, PVA particles were primarily used for tumoral bleedings. In UGIB, particulate agents and liquid embolics were generally avoided due to the presence of collateral circulation, which can make their distribution less predictable compared to microcoils. For LGIB, we predominantly used microcoils, reflecting both their safety profile and the extensive experience of our institution with these devices. The same material strategy was applied consistently for small bowel and colonic embolizations, guided by these principles.

### Definitions and data collection

Reports from TAE procedures, imaging studies, endoscopic exams, and surgical interventions were reviewed and analyzed from patients’ electronic medical records.

Technical success was defined as the cessation of bleeding on control angiography. Empiric embolization was performed when digital subtraction angiography (DSA) demonstrated neither active extravasation nor indirect signs of bleeding [10]. In these cases, the target vessel was identified based on pretreatment CTA findings or the location of endoscopic clips. For empiric embolizations, technical success was defined as the successful occlusion of the vessels presumed to be responsible for bleeding according to these guiding imaging modalities.

Clinical success was defined as the absence of rebleeding within 30 days following the procedure. In cases requiring repeat embolization, clinical success was determined based on the absence of rebleeding within 30 days of the final TAE intervention.

Thirty-day mortality was defined as any all-cause death occurring within 30 days of the TAE procedure, or within 30 days of the final TAE in patients who underwent multiple embolizations.

Disease-specific 30-day mortality was defined as death occurring within 30 days of the procedure, attributed directly or indirectly to bleeding, based on clinical assessment and, when available, autopsy findings.

Procedure-related complications were classified according to the Cardiovascular and Interventional Radiological Society of Europe (CIRSE) classification system [11].

### Statistical analysis

Analyses were conducted using SPSS 30.0. Factors evaluated for association with clinical success, 30-day mortality and disease-specific 30-day mortality included age, sex, the location of bleeding, hemoglobin level, Charlson Comorbidity Index (CCI) score, antithrombotic use, packed red blood cell (PRBC) transfusions, vasopressor/inotropic therapy, and embolization characteristics such as embolizing agents and targeted versus empiric embolizations. Multivariable logistic regression was conducted for each outcome, and statistical significance was set at *p* < 0.05.

## Results

### Baseline characteristics

The median patient age was 73 years (IQR: 16), with a gender distribution of 65 males (65.0%) and 35 females (35.0%). Of the 100 patients, 44 (44.0%) presented with UGIB, while 56 (56.0%) had LGIB. The most common etiologies were peptic ulcer disease (26/44, 65.6%) in the UGIB group and malignancy (17/56, 30.0%) in the LGIB group (Table 1). Bleeding occurred during hospitalization in 14 patients (14.0%). The most frequently embolized vascular territory was the inferior mesenteric artery (IMA) (37/111, 33.3%) (Table 2). Active extravasation or indirect signs of bleeding were identified in 79 of 111 procedures (71.2%), including 40 cases with active contrast extravasation and 39 with indirect angiographic signs of bleeding. In the remaining cases empiric embolization was performed.

**Table 1:** Etiology of Gastrointestinal bleeding by anatomical location. Data are presented as number and percentage of cases for upper (*n* = 44) and lower (*n* = 56) gastrointestinal tract bleeding

Etiology	
Upper GI tract	
peptic ulcer disease	26/44 (65.6%)
iatrogenic	4/44 (15.6%)
malignancy	9/44 (6.4%)
Mallory-Weiss syndrome	1/44 (3.1%)
erosive gastritis	1/44 (3.1%)
grafto-duodenal fistula	1/44 (3.1%)
Dieulafoy’s lesion	1/44 (3.1%)
Lower GI tract	
diverticular disease	14/56 (25.0%)
malignancy	17/56 (30.4%)
hemorrhoid	5/56 (8.9%)
other rectal pathologies	4/56 (7.1%)
iatrogenic	3/56 (5.4%)
stercoral colitis	2/56 (3.6%)
inflammatory bowel disease	3/56 (5.4%)
unknown	8/56 (14.2%)

**Table 2:** Embolized arterial territories during the procedures (*n* = 67). Data are presented as the number and percentage of cases in which each vascular territory was targeted

Embolized territory	
Gastroduodenal artery (GDA)	34/111 (30.6%)
Left gastric artery (LGA)	8/111 (7.2%)
Superior mesenteric artery (SMA)	24/111 (21.6%)
Inferior mesenteric artery (IMA)	37/111 (33.3%)
Internal iliac artery (IIA)	4/111 (3.6%)
Splenic artery (SA)	3/111 (2.7%)
Esophageal artery	1/111 (0.9%)

Most patients (82/100, 82.0%) had comorbidities, with cardiovascular disease, gastrointestinal disease, and malignancy being the most prevalent (Table 3). The CCI scores ranged from 0 to 15, with a median score of 6 (IQR: 5). A total of 51 patients (51.0%) were receiving antithrombotic therapy (Table 4).

**Table 3:** Comorbidities in the study population (*n* = 100). Values represent the number and percentage of patients with each condition. Patients May have had multiple comorbidities

Comorbidities	
Cardiovascular disease	51/100 (51%)
End-stage renal disease	8/100 (8.0%)
Gastrointestinal disease	35/100 (35%)
Liver disease	16/100 (16%)
Pulmonary disease	8/100 (8.0%)
Malignancy	48/100 (48%)
Dementia	3/100 (3.0%)

**Table 4:** Antithrombotic therapy among patients in the study population (*n* = 100). Values indicate the number and percentage of patients receiving each therapy. ASA: acetylsalicylic acid; DAT: dual antiplatelet therapy; DOAC: direct oral anticoagulant; VKA: vitamin K antagonist; LMWH: low molecular weight heparin. Some patients received combination therapies

Antithrombotic therapy	51/100 (51.0%)
ASA	15/100 (15.0%)
P2Y12 inhibitors alone	1/100 (1.0%)
DAT	8/100 (8.0%)
DOAC	12/100 (12.0%)
VKA	3/100 (3.0%)
LMWH	7/100 (7.0%
LMWH + antiplatelet	3/100 (3.0%)
DOAC + antiplatelet	2/100 (2.0%)

The mean lowest recorded hemoglobin level during hospitalization was 7.5 ± 2.0 g/dL. PRBC transfusions were required in 79 patients (79.0%), with a median of 5 units (IQR: 6.7). Vasopressors and/or inotropes were administered to 30 patients (30.0%).

Among the 44 patients with UGIB, 22 (50.0%) underwent a single attempt at endoscopic therapy, 13 (29.5%) had two attempts, and 9 (20.5%) proceeded directly to transarterial treatment. In the LGIB group, endoscopy was performed prior to catheterization in 5 cases (8.9%). Diagnostic CTA was obtained in 94 patients (94.0%). The median time between the positive CTA and catheter angiography was 2 h (IQR: 2.6).

### Embolic agents

Microcoils were the most frequently used embolic agent (89/111, 80.2%), followed by polyvinyl alcohol (PVA) particles (12/111, 10.8%) and gelatin sponge (Gelfoam) (2/111, 1.8%). Various combinations of embolic agents were used in the remaining 8 cases (7.2%) (Table 5).

**Table 5:** Types of embolic agents used during the procedures (*n* = 111). Values are presented as number of cases and percentage of total procedures. Combinations indicate use of multiple agents in a single procedure

Embolizing agents	
Microcoils	89/111 (80.2%)
PVA	12/111 (10.8%)
Gelfoam	2/111 (1.8%)
Microcoils + Gelfoam	4/111 (3.6%)
Microcoils + PVA	3/111 (2.7%)
Microcoils + Glue	1/111 (0.9%)

### Technical success

The technical success rate was 100% (111/111). All TAE procedures, including empiric embolizations, were technically successful.

### Clinical success

The overall clinical success rate of TAE was 82% (82/100), with success rates of 77.3% (34/44) in UGIB and 85.7% (48/56) in LGIB. In some patients clinical success was achieved by repeat procedure(s). A total of 10 patients without clinical success required surgery as the next therapeutic step.

### 30-day mortality

The overall 30-day mortality rate was 26.0% (26/100), with rates of 29.5% (13/44) in UGIB and 23.2% (13/56) in LGIB.

### Disease-specific 30-day mortality

Disease-specific 30-day mortality was 15.0% (15/100). Of the 26 patients who died, 11 (42.3%) passed away due to causes unrelated to GIB.

### Procedural complications

Procedural complications occurred in 4 patients (4.0%). Three cases of ischemic complications were observed in the LGIB group:

1. Ileocolic and cecal ischemia with perforation (CIRSE Grade 6).

In one patient, embolization of ileocolic branches with PVA particles for cecal bleeding with unknown etiology was followed by abdominal tenderness on post-procedural day 2. CT demonstrated non-enhancement of the cecum and terminal ileum, consistent with bowel ischemia. Laparotomy revealed necrosis of the cecum and adjacent terminal ileum, which were resected. Relaparotomy on postoperative day 3 identified anastomotic insufficiency and fecal peritonitis, requiring further resection, lavage, and ileostomy. Despite ICU management, the patient developed septic shock and died two weeks after the embolization.

2. Small bowel perforation at an anastomotic site (CIRSE Grade 3).

A second patient with a history of small bowel resection for Crohn’s disease presented with bleeding from an anastomotic site. Two weeks after superselective coil embolization, the patient was readmitted with severe abdominal pain. CT demonstrated a small bowel perforation at the anastomosis. Surgical revision with further resection and creation of a jejunostomy was performed, and the patient recovered and was discharged ten days later.

3. Localized perforation with contained abscess at the ileojejunal transition (CIRSE Grade 3).

A third patient underwent superselective coil embolization for bleeding at the ileojejunal junction. Post-procedural abdominal pain prompted CT imaging, which revealed localized ischemia with a small, contained perforation and adjacent abscess. The abscess required neither drainage nor surgical intervention, and the patient remained clinically stable. Conservative management led to gradual improvement, and the patient was discharged three weeks after the procedure.

Additionally, one patient developed an access-site hematoma that did not require invasive treatment (CIRSE grade 2).

### Determinants of clinical success

Logistic regression analysis identified vasopressor or inotropic therapy (OR: 23.75, 95% CI: 4.16–135.48, *p* < 0.001) and antithrombotic use (OR: 8.87, 95% CI: 1.51–52.09, *p* = 0.016) as significant negative predictors of clinical success. Empiric embolization was associated with lower likelihood of rebleeding (OR: 0.141, 95% CI: 0.02–0.89, *p* = 0.037) (Table 6).

**Table 6:** Variables tested for association with clinical success after transarterial embolization for gastrointestinal bleeding. Patients were stratified into successful hemostasis (n = 82) and recurrent bleeding (n = 18) groups. Data are presented as mean (standard deviation) or number (percentage). Univariate logistic regression was performed to calculate odds ratios (Exp[B]) with 95% confidence intervals (CI). CCI = Charlson comorbidity index; PRBCs = packed red blood cells; UGIB = upper gastrointestinal bleeding

	Successful hemostasis (*N* = 82)	Recurrent bleeding (*N* = 18)	*P*	Exp (B)	95% confidence interval for Exp(B)	
					Lower	Upper
Female N (%)	31 (37.8)	4 (22.2)	0.223	2.784	0.536	14.448
Age (years) mean (SD)	70.6 (14.2)	69.8 (12.4)	0.764	0.992	0.938	1.048
UGIB N (%)	34 (41.5)	10 (55.5)	0.757	1.291	0.257	6.487
Coils N (%)	66 (80.5)	16 (88.8)	0.976	1.035	0.116	9.222
Empirical embolization N (%)	25 (30.5)	3 (16.6)	**0.037**	0.141	0.022	0.890
Hemoglobin (g/dL) mean (SD)	76.2 (21.5)	72.8 (16.2)	0.788	0.995	0.957	1.034
Number of PRBCs mean (SD)	5.9 (7.0)	9.6 (8.5)	0.287	1.057	0.955	1.170
Vasopressor or inotropic therapy N (%)	17 (20.7)	13 (72.2)	**< 0.001**	23.751	4.164	135.488
Antithrombotic therapy N (%)	35 (42.7)	12 (66.6)	**0.016**	8.873	1.512	52.091
CCI mean (SD)	6.3 (3.3)	6.3 (3.0)	0.947	0.992	0.780	1.261

### Determinants of 30-day mortality

Logistic regression analysis identified vasopressor or inotropic therapy (OR: 13.48, 95% CI: 3.43–52.90, *p* < 0.001) and higher CCI scores (OR: 1.29, 95% CI: 1.06–1.60, *p* = 0.013) as significant negative predictors of survival (Table 7).

**Table 7:** Variables tested for association with 30-day mortality after transarterial embolization for Gastrointestinal bleeding. Patients were stratified into survivors (*n* = 74) and non-survivors (*n* = 26) groups. Data are presented as mean (standard deviation) or number (percentage). Univariate logistic regression was performed to calculate odds ratios (Exp[B]) with 95% confidence intervals (CI). CCI = Charlson comorbidity index; PRBCs = packed red blood cells; UGIB = upper Gastrointestinal bleeding

	Survived (*N* = 74)	Died (*N* = 26)	*P*	Exp (B)	95% confidence interval for Exp(B)	
					Lower	Upper
Female N (%)	28 (37.8)	6 (23.1)	0.571	1.443	0.406	5.129
Age (years) mean (SD)	70.2 (14.2)	71.1 (13.0)	0.791	0.994	0.947	1.042
UGIB N (%)	31 (41.9)	13 (50)	0.244	2.198	0.585	8.262
Coils N (%)	60 (81.1)	22 (84.6)	0.823	1.211	0.227	6.444
Empirical embolization N (%)	22 (29.7)	6 (23.1)	0.131	0.369	0.101	1.345
Hemoglobin (g/dL) mean (SD)	78.1 (22.3)	68.3 (12.9)	0.226	0.977	0.941	1.015
Number of PRBCs mean (SD)	5.4 (6.5)	9.7 (8.8)	0.532	1.024	0.950	1.104
Vasopressor or inotropic therapy N (%)	14 (18.9)	16 (61.5)	**< 0.001**	13.476	3.433	52.903
Antithrombotic therapy N (%)	36 (48.6)	11 (42.3)	0.590	0.721	0.220	2.362
CCI mean (SD)	5.9 (3.1)	7.3 (3.4)	**0.013**	1.298	1.056	1.597

### Determinants of 30-day disease-specific mortality

Logistic regression analysis identified vasopressor or inotropic therapy (OR: 214.14, 95% CI: 15.71–2918.21, *p* < 0.001) and higher CCI scores (OR: 1.37, 95% CI: 1.03–1.82, *p* = 0.031) as significant predictors of disease-specific mortality (Table 8).

**Table 8:** Variables tested for association with disease-specific 30-day mortality after transarterial embolization for Gastrointestinal bleeding. Patients were stratified into survivors and non-survivors according to disease-specific mortality. Data are presented as mean (standard deviation) or number (percentage). Univariate logistic regression was performed to calculate odds ratios (Exp[B]) with 95% confidence intervals (CI). CCI = Charlson comorbidity index; PRBCs = packed red blood cells; UGIB = upper Gastrointestinal bleeding

	No disease-specific death (*N* = 85)	Disease- specific death (*N* = 15)	*P*	Exp (B)	95% confidence interval for Exp(B)	
					Lower	Upper
Female N (%)	32 (37.6)	3 (20.0)	0.233	3.565	0.441	28.795
Age (years) mean (SD)	70.3 (14.0)	71.1 (13.2)	0.600	0.982	0.915	1.053
UGIB N (%)	37 (43.5)	7 (46.7)	0.070	7.460	0.846	65.769
Coils N (%)	69 (81.2)	13 (86.7)	0.796	1.439	0.091	22.675
Empirical embolization N (%)	25 (29.4)	3 (20.0)	0.058	0.156	0.023	1.065
Hemoglobin (g/dL) mean (SD)	76.3 (21.7)	71.5 (13.3)	0.842	0.993	0.927	1.063
Number of PRBCs mean (SD)	6.0 (7.1)	9.5 (8.3)	0.946	1.005	0.880	1.146
Vasopressor or inotropic therapy N (%)	17 (20.0)	13 (86.7)	**< 0.001**	214.137	15.713	2918.209
Antithrombotic therapy N (%)	39 (45.9)	8 (53.3)	0.625	1.638	0.226	11.872
CCI mean (SD)	6.1 (3.2)	7.3 (3.4)	**0.031**	1.370	1.030	1.822

## Discussion

In the present study, the technical success rate of angiographic procedures was 100%, with durable hemostasis achieved in 82% of patients. Overall 30-day mortality was 26%, and complications occurred in 4% of cases, including ischemia-related adverse events in 3%. These findings are consistent with previously published literature [12–14].

The detection rate of active bleeding on DSA in our cohort was 71%, which falls within the range reported in the literature (54–81%) [14, 15]. In cases where neither active extravasation nor indirect angiographic signs were present, empiric embolization was performed. The safety and efficacy of empiric embolization in UGIB are well established, with outcomes comparable to targeted embolization in terms of rebleeding and mortality [16, 17]. Additionally, prophylactic TAE after successful endoscopic treatment of high-risk peptic ulcers has been associated with reduced rebleeding and mortality, although this strategy is not yet incorporated into clinical guidelines [18–20]. The role of empiric embolization in LGIB remains debated. A recent meta-analysis reported similar rates of durable hemostasis for empiric and targeted embolization (23.6% vs. 21.1%), but a higher incidence of post-procedural ischemia in the empiric group (14.3% vs. 4.7%) [13]. In our study, empiric embolization was associated with a lower risk of rebleeding, while mortality was unaffected. A plausible explanation for this finding is that patients undergoing empiric embolization may have had less severe bleeding, which had already ceased by the time of angiography. With careful review of pretreatment imaging - and, in selected cases, the use of endoscopic clips for localization - we were able to effectively target the suspected culprit vessels, thereby reducing the likelihood of rebleeding in this lower-risk patient subgroup.

Another important consideration for TAE is the choice of embolic material. In most series, the preferred embolics are either coils or glue, while other commonly used options include gelatin sponge, microspheres, and PVA particles [13, 15, 21, 22]. In our study, the choice of embolic agent was made by the operator according to bleeding location, vascular anatomy, catheter position, and underlying etiology. At our institution, microcoils are the predominant embolic device used for both upper and lower gastrointestinal hemorrhage, accounting for 80% of cases, and in combination with other agents in an additional 7%. This strategy aligns with multiple contemporary reports indicating coils remain the most commonly used embolic material in clinical series. For instance, a recent meta-analysis evaluating TAE for non-variceal GIB demonstrated that glue and coils provide similarly high clinical success rates, while also highlighting that coils were the most commonly employed embolic material among the included studies [23].

In a comparative series of LGIB embolization, patients treated with coils alone had higher clinical success and lower ischemic complication rates than those receiving particles [24]. In our practice, particles are mainly reserved for the treatment of tumoral bleedings.

In our cohort, the choice of embolic agent did not significantly affect clinical success or mortality; however, this result should be interpreted cautiously due to the overwhelming predominance of patients treated with microcoils.

Patient-specific variables also influenced outcomes. Higher CCI scores were strongly associated with increased mortality, echoing findings from studies on general GIB populations [25–27], but our study is among the first to demonstrate the magnitude of this association specifically in patients undergoing TAE. This relationship is clinically plausible: a substantial proportion of deaths (42%) were not directly attributable to bleeding, suggesting that comorbidity burden - rather than hemorrhage severity alone - played a decisive role in outcomes. GIB is well recognized to occur as part of the end-of-life trajectory in frail, multimorbid patients, who are often poor surgical candidates and present limited physiological reserve even when bleeding is controlled [28]. Thus, in this high-risk subgroup, TAE may successfully arrest hemorrhage, yet mortality remains driven by underlying disease processes. Despite the unfavorable prognosis associated with advanced comorbidity, current clinical practice standards do not support leaving GI hemorrhage untreated; therefore, TAE is often pursued even when the underlying disease burden limits the likelihood of survival.

The most significant predictor of both clinical failure and mortality was the need for vasopressor or inotropic support. These patients experienced markedly higher rebleeding and mortality rates. Vasopressor requirement reflects profound hemodynamic instability in a subgroup with severe bleeding, in whom fluid resuscitation alone is insufficient to restore adequate perfusion. Consequently, the poor outcomes associated with vasopressor use likely stem from the underlying severity of shock physiology rather than the pharmacologic agents themselves. At the same time, the vasoconstrictive effects of these drugs may reduce splanchnic blood flow, thereby lowering angiographic sensitivity, hindering catheterization, and masking active bleeding during TAE. Once vasospasm resolves, previously undetected bleeding vessels may become reperfused, contributing to the higher risk of rebleeding. These combined factors may explain why vasopressor use predicted not only mortality but also lower clinical success rates [29].

Antithrombotic therapy also negatively affected clinical success. Similar to vasopressors, antithrombotics have been associated with increased rebleeding risk [30]. Some studies suggest that glue may be more effective in this subgroup due to its coagulation-independent mechanism of action, though this may be offset by a higher rate of ischemic complications [31, 32].

Interestingly, neither the lowest recorded hemoglobin level nor the number of PRBC transfusions predicted rebleeding or mortality in our cohort. Although current guidelines recommend a restrictive transfusion strategy, this approach remains debated [27, 33–36]. Some studies have reported that the number of transfused units is an independent predictor of mortality, whereas others have found no such association.

Based on existing literature, other potential confounders affecting patient outcomes include coagulopathy and time to intervention [13–15]. In the present study, coagulation status was routinely monitored and corrected when necessary, in accordance with our institutional protocol. Unfortunately, reliable data on the time elapsed between symptom onset and TAE treatment were not available. The median time from positive CTA to catheter angiography was 2 h, indicating a potential area for process improvement.

This study has several limitations. The most important is its retrospective design, which may introduce selection bias. Additionally, the moderate sample size may limit statistical power and increase the risk of overfitting in multivariable analyses, rendering the findings exploratory. The limited patient number also precluded separate analysis of predictors for UGIB and LGIB. Long-term follow-up outcomes were not assessed; however, given the advanced age and comorbidities of our patient population, we believe these outcomes may have limited clinical relevance. We did not compare TAE outcomes with other treatments such as endoscopic therapy or surgery, which reflects the challenges of conducting comparative studies in this clinical setting. However, as TAE indications were based on established clinical guidelines, our results represent real-world practice. Lastly, although some predictors such as vasopressor use emerged as significant, residual confounding from unmeasured factors cannot be excluded.

## Conclusions

In accordance with previous reports, TAE demonstrated high technical efficacy, along with favorable clinical outcomes and a satisfactory safety profile, in the treatment of nonvariceal GIB. Vasopressor or inotropic treatment, antithrombotic use, comorbidity burden, and empiric embolization were all associated with differences in outcomes. Among these, vasopressor requirement emerged as the strongest predictor of both rebleeding and mortality; however, this association likely reflects the underlying severity of hemodynamic instability in these critically ill patients rather than the direct effect of vasopressor therapy itself. These findings underscore the need for further prospective studies to evaluate optimal treatment strategies in patients presenting with severe shock physiology who require vasopressor support.

## Data Availability

The datasets used and/or analysed during the current study are available from the corresponding author on reasonable request.

## Abbreviations

GIB: Gastrointestinal bleeding
TAE: Transarterial embolization
UGIB: Upper gastrointestinal bleeding
LGIB: Lower gastrointestinal bleeding
ACG: American College of Gastroenterology
INR: International normalized ratio
CTA: Computed tomography angiography
DSA: Digital subtraction angiography
CFA: Common femoral artery
IMA: Inferior mesenteric artery
GDA: Gastroduodenal artery
CIRSE: Cardiovascular and Interventional Radiological Society of Europe
CCI: Charlson Comorbidity Index
PRBC: Packed red blood cell
PVA: Polyvinyl alcohol
